# Medication related to pigmentation of oral mucosa

**DOI:** 10.4317/medoral.25110

**Published:** 2022-04-14

**Authors:** María del Carmen Mallagray-Montero, Luis Alberto Moreno-López, Rocío Cerero-Lapiedra, María Castro-Janeiro, Cristina Madrigal-Martínez-Pereda

**Affiliations:** 1 Departamento de Especialidades Clínicas Odontológicas. Facultad de Odontología, Universidad Complutense de Madrid

## Abstract

**Background:**

The diagnosis of oral melanotic lesions is, more often than not, challenging in the clinical practice due to the fact that there are several reasons which may cause an increase in pigmentation on localized or generalized areas. Among these, medication stands out.

**Material and Methods:**

In this work, we have carried out a review in the reference pharma database: Micromedex® followed by a review of the scientific published literature to analyse coincidences and possible discrepancies.

**Results:**

Our findings show that there are several prescription drugs that can cause pigmented lesions in the oral mucosa. This must be known by clinicians in order to properly diagnose pigmented lesions. We have identified a set of 21 medicaments which cause these lesions, some of which are used frequently in the clinic, such as Metronidazole, Amitriptyline, conjugated oestrogens and Chlorhexidine gluconate. We also found discrepancies with the data published in specialized literature, some of which wasn’t reflected in the Summary of Product Characteristics.

**Conclusions:**

Our work highlights the importance of the proper communication of adverse drug reactions (ADR) by health professionals in order to provide thorough and accurate information and diagnosis.

** Key words:**Adverse drug reactions, oral pigmentation, micromedex.

## Introduction

The term Adverse Drug Reactions (ADR) was defined in 1972 by the World Health Organization (WHO) but has since undergone two modifications, first in 1995 and then in 2007 after which it was concluded that the term ADR should also include the involuntary and harmful effects derived from medication errors as well as uses beyond commercialization (1-3).

ADR constitute the most frequent complications related to the use of medication and are a major cause of morbidity and mortality (4). In fact in 2007 the WHO included ADR in the list of the ten leading causes of death worldwide (5).

Pigmented lesions of the oral mucosa are areas that have undergone changes in their physiological coloration due to the deposit of endogenous or exogenous pigments. The aetiology of these lesions is diverse and undetermined in some cases as they may arise by a physiological, reactive, or neoplastic mechanism, as well as be part of a systemic disorder or even be idiopathic (4).

**Figure 1:**
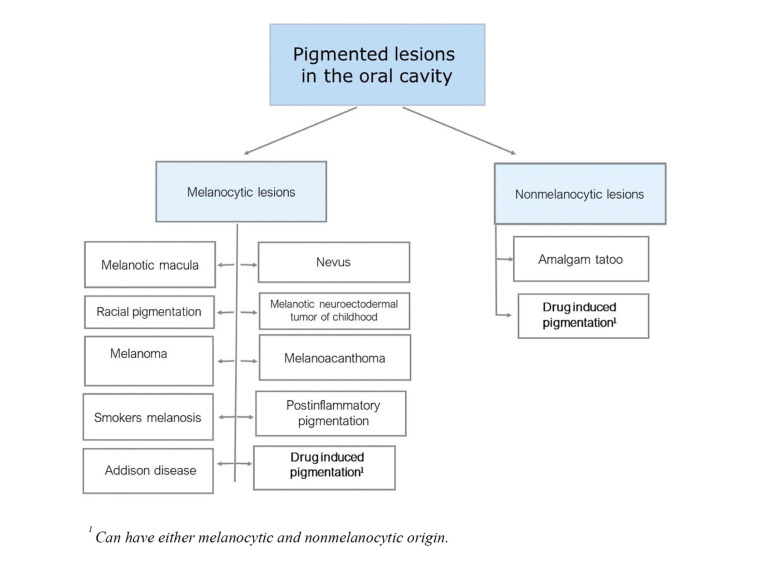
Classification of the most frequent melanocytic lesions according to their aetiology (modified from Tavares *et al*.).

The pigment accumulated in these lesions can have an intrinsic, as is the case of melanin, or extrinsic origin, which on the other hand are formed by the accumulation of exogenous substances. Melanocytic lesions appear due to an increase in melanin production and, less frequently, due to an increase in the number of melanocytes themselves. Additionally, the cause of this accumulation can have an endogenous or exogenous origin (6,7). Thus, pigmented lesions can be either endogenous or exogenous in origin and the pigment itself can be intrinsic (melanic) or extrinsic (non melanic), as shown in Fig. 1 (8).

The aforementioned lesions are generally flat or macular. They can either be localized or diffuse and the pigmentation can vary from brown to grey, blue, and even black. Due to the similarities between these lesions it is of the utmost importance to avoid diagnosis based solely on clinical characteristics as this could lead to an erroneous diagnosis (8). Moreover, a histopathological analysis to confirm the diagnosis may be necessary in some patients, since some melanotic lesions can be malignant (6).

Pigmented lesions constitute approximately 2% of the diagnoses made through biopsy in the oral cavity. In most cases, the clinician is dependent on the information on the patient’s medical records to make an accurate diagnosis and find the underlying cause of pigmentation, since neither the appearance nor the histopathological analysis are normally sufficient to make an accurate diagnosis (6,7).

Oral pigmentation induced by prescription drugs can be either melanocytic or non-melanocytic in origin, Fig. 1. Regardless of the origin, hyperpigmentation induced by medication usually causes a widespread change of colour which is in contrast with the physiologic pigmentation (8). This has been widely documented in publications that highlight the cause-and-effect relationship between the administration of a specific drug and the appearance of pigmented lesions in the oral cavity (9). These lesions may appear immediately after the administration of a medicine or after a longer period such as days or even years (10).

In order to make a proper diagnosis, it is important to know if these mucosal areas with a change in colouring have arisen as an ADR to a certain medication. Patients presenting these lesions must be monitored and a proper follow-up of the lesions needs to be done in order to avoid misdiagnosis. In addition, a correct record of the ADR in the oral cavity in response to the prescription needs to be made (9,10).

The information about ADR is registered in the Summary of Product Characteristics (SPC), and can be consulted in different sources. For instance in Spain, through the Spanish Medicine and Medical products Agency (Agencia Española de Medicamentos y Productos Sanitarios, AEMPS) on its website under the Online Center of Information about Medicines (Centro de Informacion del Medicamento de la AEMPS, CIMA) or through the system of Pharmaceutical Surveillance on Adverse Reactions Suspicion (Farmacovigilancia Española de Datos o de las sospechas de Reacciones Adversas, FEDRA).

Another non-national source would be through public access drugs databases or scientific databases. Among the first one can find Micromedex® (IBM MICROMEDEX IBM Watson Health products Corporation 2020), a tool widely used for the medical management of prescription drugs. Micromedex® consists of a set of medical, pharmacological and toxicological information databases. In this publication this database was used because it allows to identify contraindications, ADR reactions and incompatibilities between different drugs and pathologies in a clear and prompt manner. The latter category of databases includes Pubmed and WOS (Web of Science), scientific databases which are not specific to drug management.

- Rationale and objectives

Health professionals have a legal obligation to notify every suspicion of ADR. However, these reactions are usually mild and it is possible some go unnoticed or unreported. Therefore, their study is often through the publication of clinical cases or series thereof. There is no review which provides a specific and systematic research of ADR compared with the published cases, to the authors’ knowledge.

This paper aims to identify those drugs in whose SPC there is evidence of a relationship with melanotic lesions in the oral mucosa.

Additionally, we aim to verify that the data registered in the SPC match the information available in the scientific literature.

## Material and Methods

The Micromedex® database was selected for this study based on the criteria established by Rodriguez-Terol in 2008 (11), namely that it is an international database, publicly available, known by health professionals and which has been referenced in several papers (12-14).

The search strategy was defined following the User’s Guide instructions (IBM Micromedex® User Guide). The instructions specified that in order to enable the identification of drugs that cause a specific ADR, the search should include the clause: ”Drugs that cause…”, together with the ADR itself. This clause was thus coupled with the terms “Hyperpigmentation”, “Discoloration”,” Staining”, “Spots”, “Melanosis” “Tanning” and “Colour change”. The results were analysed and curated, selecting only those which applied to the oral mucosa, excluding other extraoral localizations as well as the hard tissues of the oral cavity. To this end, a list including the prescriptions related to the search terms used was obtained. Then, after a thorough review of the ADR described in each of them, the ones which described hyperpigmentation or any of its synonyms in the hard tissues of the oral cavity (teeth) or not within the oral cavity were discarded. Lastly, duplicated medicines were excluded.

The most important characteristics related to the ADR caused by each drug were described considering the following parameters:

1) Frequency of appearance

2) Localization

3) Colour

4) Size

5) Duration

6) Drug administration route

A search was then carried out in Pubmed and WOS focusing on each of the prescription drugs identified, to confirm the data compiled using Micromedex®.

## Results

**Figure 2:**
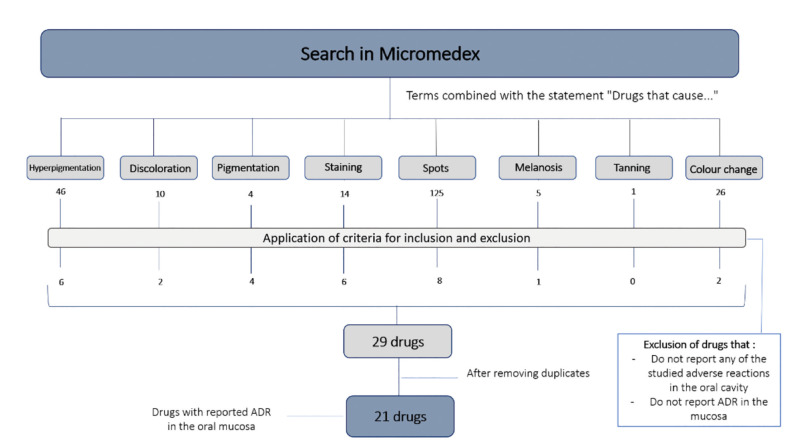
Search strategy for medication related to oral pigmentation for Micromedex® database.

Results obtained according to the search strategy are shown in Fig. 2. The number of hits per term used in the search was as follows: Hyperpigmentation 46, Discoloration 10, Pigmentation 4, Staining 14, Spots 125, Melanosis 5, Tanning 1, and Colour change 26. Only those relevant to the mucosa were selected, excluding other extraoral localizations as well as the hard tissues of the oral cavity. This led to a selection of a total of 29 drugs which were further reduced to 21 after excluding duplicates (Fig. 1). These prescription drugs were then classified according to therapeutic families as shown in Table 1. The characteristics of the ADR themselves are described in Table 2.

We have found scientific reports supporting Micromedex® results for every medicament.

The results show that for 16 out of the 21 medicaments selected the report was of generalized lesions, for 4 localized lesions were reported and there was 1 in which the lesion reported evolved from focal to generalized. Regarding localization the most frequent is the tongue (11 drugs) with the dorsum being the most prevalent area, whereas the lateral borders of the tongue are only related to hydroxyurea. The buccal mucosa is the second most frequent localization followed by the gum, hard palate and lips. The floor of the mouth is the least frequent location, damaged only by conjugated oestrogens.

**Table 1:**
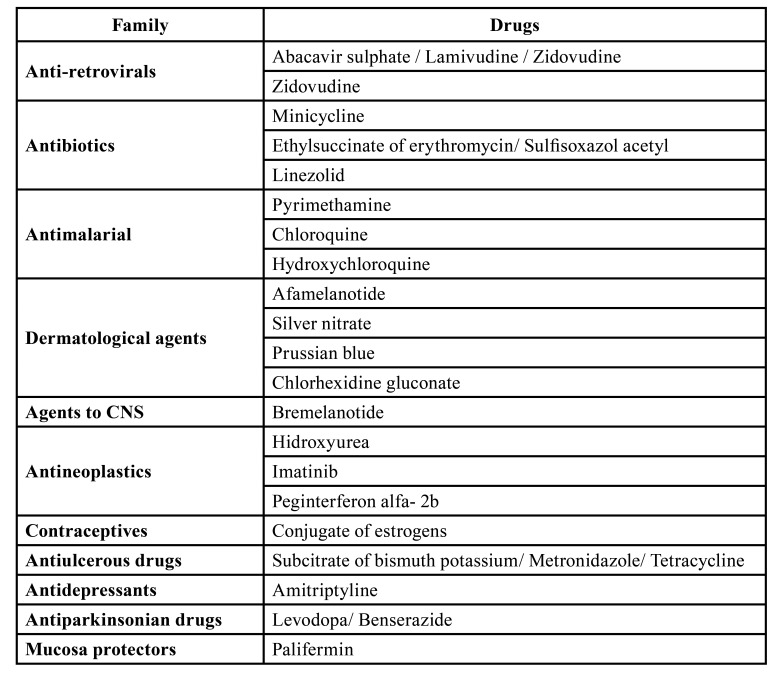
Classification of prescription drugs according to their pharmacological family.-

**Table 2:**
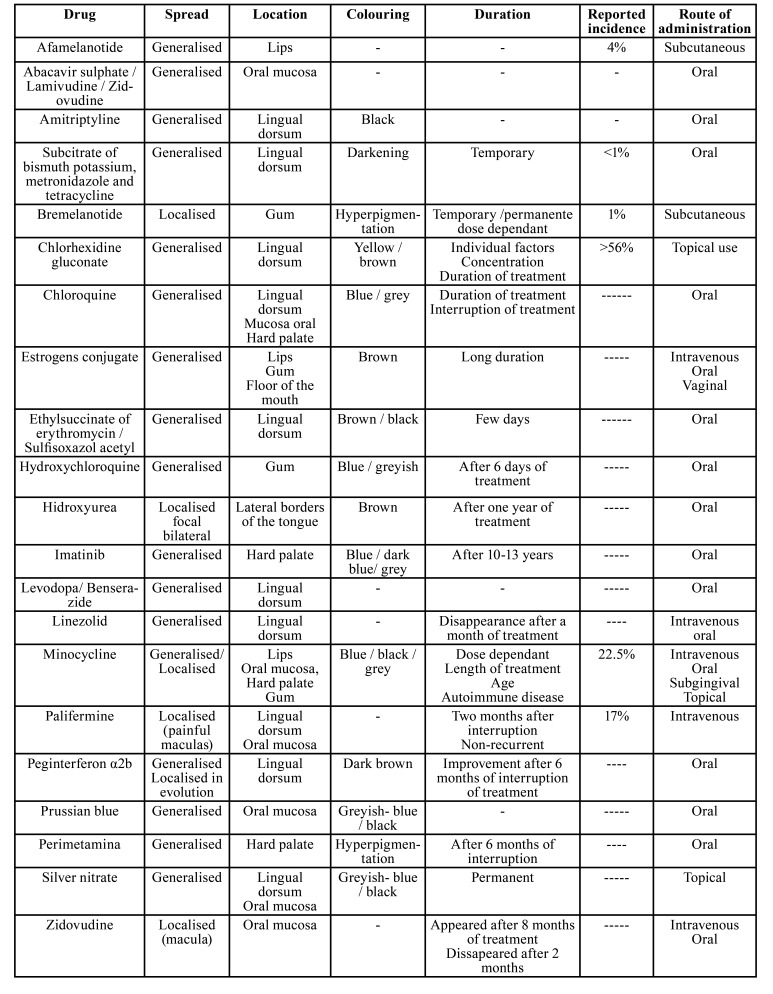
Description of the clinical variables related to melanotic lesions produced by prescription drugs.

On the other hand, colouring is not specifically notified in some of the reports, whereas for others it is described as blue, brown, grey, black, just darkening, hyperpigmentation or even yellow.

The duration of the lesions depends on each prescription and its mechanism of action. For most of the prescriptions it is dose-dependent. Evidence of disappearance of the lesions after the withdrawal of the medication causing them has not been found.

## Discussion

There are many medication databases in different formats, open access as well as behind a paywall. Nevertheless, in the clinical practice they are seldom used, which is not further aided by the fact that they are sometimes complicated to navigate. This is not only due to the fact that there is a great number of databases and information sources available but also to the discrepancies between the information they provide and the quality of the databases themselves (12,13).

CIMA was chosen as a good candidate for the search, as it is the official source of information on medicaments in Spain, however we found a limitation, namely that the search is limited to the prescriptions whose SPC has been fractioned by the laboratory in the format required by the CIMA website. This information is not available for many medications.

Thus, before we could start our search we needed to find a database which not only allowed to identify medicaments which cause a specific ADR, but which also classified the ADR according to its severity and amount of evidence supporting it and furthermore allowed to contribute bibliographic references and a description of the clinical handling (11).

These guidelines were introduced by Rodriguez-Terol *et al*. in 2008 when they conducted a research work aiming to identify available databases which deal with pharmacological interactions and assess their quality. We selected Micromedex® since it enabled us to carry out this screening according to the ADR of our choice, pigmented lesions (11).

These pigmented lesions can appear, as mentioned earlier, immediately after the administration of a specific medication, after just one dose or after having taken it for several days or years (8). Currently, the mechanism that causes the pigmentation is unclear, but it is believed that it could be due to an increase in the number of melanocytes in the tissue, an increase in melanin synthesis or deposit of metabolites derived from the prescription drugs in the tissue (9). Some medications may also generate a change in the colouring of the hard tissues such as the alveolar bone or the tooth and it has been observed that the appearance of each type of lesion depends on the type of prescription drug that has caused it, suggesting different action mechanisms. The published literature records antineoplastic drugs as the most frequent pharmacological family related to the appearance of melanocytic pigmentation, followed by antimalarial medicines (15).

These lesions may appear in the mucous membranes in a focal or multiple way, localised or diffuse. After analysing the corresponding published literature, we confirmed that the locations where these pigmentations appear most frequently are the hard palate, gums and buccal mucosa. This is in agreement with our findings (16,17).

Most prescription drugs identified present a dose-related relationship with the intensity and extension of pigmentation. Lesions usually disappear when the administration of the medication is interrupted. In the literature we found cases of lesions that did not disappear after the interruption of the pharmacological therapy in patients treated with minocycline, imatinib and hydroxychloroquine (18-20)

Concerning the extension of the pigmented lesions, we can conclude that the pigmentation of the mucosa is more often generalised than focal. The descriptions of the colour of the lesions are subjective and do not enable us to draw clear conclusions about the prevalence of one colour or another.

In the systematic review based on case reports and series thereof published in 2020 by Binmadi *et al*. (10) they reported that the medication that was more frequently associated with these pigmentations is imatinib. The hyperpigmentation caused by this prescription has been described in several studies, such as the ones published by Mcpherson *et al*. 2009 (21), Dai *et al*. 2017 (22), Steele *et al*. 2012 (23), all of which concluded that the duration of the treatment was the main risk factor for the formation of bigger and darker hyperpigmented lesions.

Imatinib causes a well-defined blueish to greyish pigmentation on the hard palate. Its action mechanism is based on the inhibition of the channel c-KIT, involved in the development of melanocytes and their regulation. This also explains why histologically, the oral mucosa in these patients presents a deposit of melanocytes on the lamina propria (24).

As mentioned before the literature mentions larger and darker lesions in correlation with the duration of the administration (21-23).

Another antineoplastic drug identified is hydroxyurea. There are published reports confirming the relationship between the oral pigmentation and the administration of this drug. The most usual location is the tongue (25).

Regarding antimalarial drugs, the pigmented lesions related to chloroquine and hydrochloroquine correspond to diffuse blue to grey areas located in the hard palate and the buccal mucosa. In some cases this pigmentation has been reported to spread to the lingual side and gingival margin. The biopsies performed on these patients reveal deposits of dark brown granulated pigments in the lamina propria, with infiltration of fibroblasts and macrophages in the subepithelial and perivascular areas. This hyperpigmentation of the mucosa is reverted when the therapy with the antimalarial drug is reduced or interrupted. A long-lasting administration (up to 15 years) of antimalarial drugs was associated to serious diffuse blueish to grey or black lesions on the hard palate with cutaneous alterations (26-28). It is worth noting that the use of these medications in the treatment of Sars-CoV-2 will shed more light on this matter in the future, after a follow- up of the affected population (29).

Other medications identified by the search which coincided with those mentioned in the literature are minocycline and zidovudine. Minocycline is an antibiotic that produces hyperpigmentation in the hard palate, buccal mucosa, sublingual area and gum. Greyish patches in the dorsum of the tongue also appear. In the clinical studies we have analysed, patients who presented pigmentation associated to minocycline had undergone a long-term therapy and the pigmentation disappeared after having interrupted the treatment for six months (15).

Zidovudine is an anti-retroviral treatment which produces pigmentation six to eight months after treatment onset. The associated lesions consist of a brownish patch or stain in the buccal mucosa and lips (30). These lesions disappeared roughly two months after the interruption of the treatment. It is thought that its mechanism of action could be due to an increase in the production of melanin as there is an increase in the secretion of the melanocyte-stimulating hormone (31). Another antimicrobial drug identified in our search is pyrimethamine, an anti-protozoan used to prevent malaria, for which some cases associated to hyperpigmentation in areas of the palate have been published (32). In our search in Micromedex® we also found the combined treatment of abacavir sulphate/lamivudine/zidovudine as a cause of oral pigmentation.

Among the dermatological treatments, there is evidence that afamelanotide (used in the prevention of phototoxicity in patients with erythropoietic porphyria) produces pigmentation in the mucosa as a side effect, which is registered as an ADR. Its mechanism of action is caused by an increase in the number of melanocytes in the tissue, resulting in brownish melanocytic lesions (33).

Conversely, other medications such as chlorhexidine, prussian blue or silver nitrate cause hyperpigmentation due to the breakdown of the pigmented metabolites which constitute them. These changes in colouring of the mucosa are reversible.

In our search we found that the only pigmentations whose course includes painful symptoms are the ones caused by palifermin. The hyperpigmentation this prescription drug produces appears as round-shaped lesions restricted to the tongue and buccal mucosa, and the pain associated to it usually disappears two days after its onset. The pigmented lesions disappear 20 days later.

There are drugs identified in systematic reviews as inductors of oral pigmentation, but they do not have this ADR registered in their SCP and that is the reason why we could not find them in our search. Among them we can highlight: golimumab, whose SCP does not mention hyperpigmentation neither in the oral cavity nor in the skin (15); ketoconazole, not described in any SPC (10); amlodipine, whose SPC only describes change of pigmentation on the skin (34); retigabine, an antiepileptic drug which has not been commercialised in Europe since 2018; clofazimine, an antileprotic whose SPC is only associated with skin lesions.

All of the above indicates that there is a certain lack of registering pigmented lesions as an ADR through the official channels, perhaps due to their limited clinical significance. This could interfere with the proper update of the SPCs and reduce the available information regarding ADR.

## Conclusions

It is important to consider that patients with hyperpigmented lesions as ADR to a drug must be monitored for an accurate diagnosis and evaluation of possible changes in the lesion; despite the fact that so far none of these lesions have become malignant.

Micromedex® is a useful tool for identifying drugs according to their ADR, even though the search algorithm presents limitations since it does not allow a combined search of different terms, thus impeding the identification of the SPCs of certain medications. It would be highly desirable for the CIMA to have a comprehensive ADR search application.

With the aim of performing an accurate diagnosis of the pigmented lesions of the mucosa in mind, it is crucial to know which drugs may cause them and therefore all clinicians should accurately report the ADR to any given medication.
